# Alarming findings about genomics of sudden cardiac arrest in India

**DOI:** 10.1186/1755-8166-7-S1-P102

**Published:** 2014-01-21

**Authors:** Pankaj Mankad, Srisanth Balan, Saleem Mohammed

**Affiliations:** 1 Xcode Life Sciences, Chennai, 600 034, Tamil Nadu, India

## Background

Over 10% of total deaths and 50% of cardiac deaths in India are Sudden Cardiac Deaths (SCA) and occur at a young age of 5-8 years. We analysed risk allele plus genotype frequencies and polymorphism associations of 38 SNPs, confirmed in GWAS studies that increasing the risk of SCA in 124 random people in the general population. The data were compared with Caucasians reported in HapMap (n = 113).

## Material and methods

Following DNA extraction from saliva, genotyping was performed using Illumina golden gate genotyping assay. Allele and genotype frequencies were determined by direct gene count method.

## Results

Population Genomics indicated Odds Ratio of risk alleles as 1.62 (1.31 -1.92). The carrier rate of risk allele frequency was significantly higher (p = 0.037 to p = 9.18E-37) in Indians compared to Caucasians in 56% of SNPs in H-W equilibrium (15 of 27).6 SNPs showed statistically insignificant comparatively lower risk allele frequency in Indians. Mean increase in higher risk allele frequency SNPs was 2.4 fold (1.1 – 6.7). Frequency in Indians, for the two SNPs with the strongest SCD risk correlate in the gene BAZ2B was 2 times (rs4665058) and 6 times (rs 174230) greater than in Caucasians.

Personalized Genomics indicated that even the frequency of risk homozygous genotype, resulting in the SCA threat in affected individuals being the square of the risk allele hazard, was markedly different with 4.7% Caucasians carrying homozygous risk genotype versus 10.6% of Indians (p<.0001). In 18 (out of 38) SNPs studied, the homozygous genotype difference in Indians was more than double). Although interplay of gene-gene interaction in individualized risk prediction is still undefined, 59% (73 of 124) people carried between 10 to 19 risk alleles and 41% (51 of 124) had between 20 to 30 risk alleles (median 19, range 10 -25), of the total maximum of 76.

## Conclusions

This is the first report, highlighting extremely high genomic propensity for Sudden Cardiac Arrest in Indians compared to the Caucasians. In India, there is also a high propensity for risk towards homozygous genotype and people carrying several risk alleles for SCA. At the population level, this data reinforces an urgent need to train community in different locations in basic resuscitation skills and at a personal level, it raises an important point to test the vulnerable group for assessing the risk alleles for better individualized preventive action plans could be advocated.

